# Effects of Gold Nanoparticles on the Response of Phenol Biosensor Containing Photocurable Membrane with Tyrosinase

**DOI:** 10.3390/s8106407

**Published:** 2008-10-16

**Authors:** Sharina Abu Hanifah, Lee Yook Heng, Musa Ahmad

**Affiliations:** School of Chemical Sciences and Food Technology, Faculty of Science and Technology, Universiti Kebangsaan Malaysia, 43600 Bangi, Selangor, Malaysia; E-mails: shenna81@hotmail.com (S.A. H.); andong@ukm.my (M. A.)

**Keywords:** Hydrophilic polymer, methacrylate, acrylate, tyrosinase, gold nanoparticle, phenol biosensor

## Abstract

The role of incorporation of gold nanoparticles (50-130 nm in diameter) into a series of photocurable methacrylic-acrylic based biosensor membranes containing tyrosinase on the response for phenol detection was investigated. Membranes with different hydrophilicities were prepared from 2-hydroxyethyl methacrylate and n-butyl acrylate via direct photocuring. A range of gold nanoparticles concentrations from 0.01 to 0.5 % (w/w) was incorporated into these membranes during the photocuring process. The addition of gold nanoparticles to the biosensor membrane led to improvement in the response time by a reduction of approximately 5 folds to give response times of 5-10 s. The linear response range of the phenol biosensor was also extended from 24 to 90 μM of phenol. The hydrophilicities of the membrane matrices demonstrated strong influence on the biosensor response and appeared to control the effect of the gold nanoparticles. For less hydrophilic methacrylic-acrylic membranes, the addition of gold nanoparticles led to a poorer sensitivity and detection limit of the biosensor towards phenol. Therefore, for the application of gold nanoparticles in the enhancement of a phenol biosensor response, the nanoparticles should be immobilized in a hydrophilic matrix rather than a hydrophobic material.

## 1. Introduction

Electrochemical methods based on enzymes have been widely used for the measuring of phenolic compounds because of the advantages of good selectivity, long-term stability and potential for miniaturization and automation [1]. Compared to free enzyme in solution, the immobilized enzyme is more stable and resistant to various environmental changes [2]. Some authors have reported immobilization via entrapment of redox proteins or enzymes, including tyrosinase in membranes consisting of poly(vinyl pyridine), poly(vinyl imiazol), poly(acrylic acid) or poly (allyl amine) [3]. Tyrosinase, a monophenol mono-oxygenase enzyme, which catalyzes the oxidation of the phenol group to o-quinone [4] is commonly used in the detection of phenolic compounds.

The use of gold nanoparticles immobilized together with an enzyme in an electrode membrane have been shown to improve the response of the enzyme electrode where gold nanoparticles of small size allow more freedom in the orientation for the anchored protein molecules and hence maximize the utilization of their bioactive sites [5, 6]. Immobilization of gold nanoparticles together with redox enzymes for electrochemical biosensors construction provides several advantages as far as biosensor response is concerned. The immobilization of a redox enzyme together with colloidal gold is thought to either help the protein to assume a favourable orientation or to make possible conducting channels between the prosthetic groups and the electrode surface; both reducing the effective electron transfer distance, thereby facilitating charge transfer between the electrode and the enzyme [7]. Gold nanoparticles have large surface areas and good electronic properties [8] and they provide a stable surface for enzyme immobilization and allow the electrochemical sensing to be performed without the need of external electron-transfer mediators. They can act as tiny conduction centers to facilitate the transfer of electrons. So it has been used for the study of the direct electron transfer of proteins [9]. As such they can also act as electrodes of nano-size that electrically communicate between enzymes and bulk electrode materials. Because of the gold surface permits absorption of protein molecules, gold nanoparticles have been used as a matrix for enzyme immobilization where the bioactivity of macromolecules is retained. The good conductivity properties of nanoparticles enable the design of simple, sensitive and stable electroanalytical devices based on enzyme immobilization [4].

In this paper, we report the use of gold nanoparticles to enhance the response of phenol detection by biosensor constructed from the immobilization of tyrosinase in a series of methacrylic-acrylic type membranes of different hydrophilicities. These membranes, prepared by a direct and simple photocurable procedure consists of copolymers of 2-hydroxyethyl methacrylate (HEMA) and poly(2-hydroxyethyl methacrylate-co-n-butyl acrylate) with the HEMA and n-butyl acrylate (nBA) in different compositions to alter the hydrophilicity of the membrane. The objective is to examine the effects of various membranes containing immobilized tyrosinase and gold nanoparticles under different membrane hydrophilicities on the response of electrochemical biosensors for phenol detection.

## 2. Methodology

### 2.1 Reagents

Tyrosinase (EC 1.14.18.1, 2870 U/mg solid from mushroom), monomer 2-hyroxyethyl methacrylate (HEMA) and n-butyl acrylate (nBA) were purchased from Sigma. The photoinitiator 2, 2-dimethoxy-2-phenylacetophenone (DMPP), phenol and gold nanoparticles (50-130 nm) were obtained from Aldrich. The supporting electrolyte was 0.05 M phosphate buffer, prepared from potassium dihydrogen phosphate and potassium chloride that were purchased from Systerm and Merck respectively.

### 2.2 Apparatus and measurements

Amperometric experiments were carried out in a stirred electrochemical cell containing 5 ml of 0.05 M phosphate buffer/0.1 M KCl using an Autolab PGSTAT 12 Potentiostat. The working electrode was a carbon paste screen-printed electrode coated with tyrosinase containing photocured methacrylic-acrylic copolymer film. Ag/AgCl (3 M KCl) (Orion) and a glassy carbon electrode (Methrom) were used as reference electrode and counter electrode respectively. The amperometric measurements for phenol were performed at -0.10 V versus Ag/AgCl reference for electrode based on photoHEMA membrane while -0.15 V for electrodes based on photoHB91 and photoHB82 membranes.

### 2.3 Preparation of phenol biosensor

Biosensor for phenol was fabricated based on the immobilization of tyrosinase in various types of methacrylic-acrylic membranes with varied hydrophilicity. For the most hydrophilic membrane, 100% of HEMA monomer was used. For less hydrophlilic membranes, 90% of photoHEMA and 10% of nBA monomers (w/w) (photoHB91) or 80% of HEMA and 20% of nBA monomers (w/w) (photoHB82) were prepared. For photocuring purpose, 1.6% (w/w) of photoinitiator DMPP was added to the monomer mixtures. The enzyme tyrosinase with a concentration of 18.5 mg/mL (53.1 U/mL) was prepared in 0.05 M phosphate buffer/ 0.1 M KCl at pH 7.0. The final membrane cocktail was obtained by mixing 10μl of the monomer mixture and 10μl of tyrosinase enzyme solution. Gold nanoparticles (50-130 nm) in the following amount: 5.0 mg, 1.0 mg and 0.1 mg were added to the membrane cocktails before photocuring. The photocuring of the final cocktails was performed under ultra-violet (UV) radiation in a UV box (RS Ltd.) containing four 60 watt UV lamps. The irradiation was carried out for 5 min under a nitrogen atmosphere. A rigid and thin (100 μm) polymer film coated on the screen printed electrode was obtained after exposure to the UV light.

The investigation of the water absorption characteristics of various membranes was carried out by exposing membranes to 0.05 M phosphate buffer. The changes in weight of the membranes were then recorded every five min and the percentage of water content and absorption were later calculated.

### 2.4 Electrochemical measurements

The determination of phenol concentration was carried out electrochemically by measuring the current that corresponded to the electrochemical reduction of the enzymatically generated quinone. This was carried out by immersing the working, reference and counter electrodes in a 0.05 M phosphate buffer/ 0.1 M KCl at pH 7.0 under constant stirring in a electrochemical cell. A potential of -0.10 V versus Ag/AgCl (3 M KCl) was used for electrode with photoHEMA membrane whilst -0.15 V was for electrodes with photoHB91 and photoHB82 membranes. After the background current had become steady, standard solutions of various concentrations of phenol were added to the electrochemical cell and the change in current was recorded. The limit of detection of the phenol biosensor was determined by using five different screen printed electrodes where their blank response was determined. From this blank response, the detection limit was then calculated based on the average of the blank signal plus three times its standard deviation [10]. The response times of all biosensors were recorded when the biosensor reached 95% of steady state current.

## 3. Results and Discussion

### 3.1 The linear response slope of the phenol biosensor

The response slope of phenol biosensor with the three types of methacyrlic-acrylic membranes containing fixed amount of immobilized tyrosinase and varied amounts of gold nanoparticles are listed in Table 1. For biosensor with photoHEMA membrane, addition of various amounts of gold nanoparticles did not seem to affect the response slope. But for biosensors with photoHB91 or photoHB82 membranes, a marked decrease in the values of the response slopes was observed, especially for the least hydrophilic membrane photoHB82 where the reduction in the response slope was almost 33 times. There is a general trend of decreasing of the values of the response slopes from the most hydrophilic membrane photoHEMA (approximately 20% equilibrium water absorption) to the least hydrophilic photoHB82 (approximately less than 7% equilibrium water absorption) when gold nanoparticles were added.

The observed decreasing effect on the linear response slope of each biosensor towards phenol appears to be related to the decreasing of the hydrophilicity of the membrane, this is particularly obvious for HB82 membrane where it has the highest amount of the ‘hydrophobic monomer’ nBA and thus lowest hydrophilicity. The deterioration of the sensitivities of the biosensors with less hydrophilic membranes is a result of the poor diffusion process that occurred within these membranes. Studies on the diffusion properties in various photoHEMA membranes containing increasing amount of a hydrophobic monomer like methyl methacrylate had demonstrated reduction in the values of diffusion coefficients when these methacrylic type membranes, which were used for a glucose biosensor became more hydrophobic [11, 12]. With the increasing difficulty in diffusion of the hydrophilic phenol in the more hydrophobic matrices, addition of gold nanoparticles further hinders the diffusion process by creating hurdles for the substrate to reach the enzyme. Thus, the observed decrease in sensitivity is a combination effect from both loss of hydrophilicity and obstruction by gold nanoparticles in the membrane. For membrane HB82 where the hydrophilicity is the lowest, the loss in sensitivity is thus the most severe. However, such sensitivity is still comparable with several reported phenol biosensors, e.g. Wang & Dong [13] using silica sol-gel membrane on glassy carbon electrode (0.0024 μA/μM) or Sanz, *et al.* [4] with ruthenium on carbon paste electrode (0.0008 μA/μM).

### 3.2 Effets of gold nano-particles on the detection limit of the phenol biosensors

**Figure 1.**
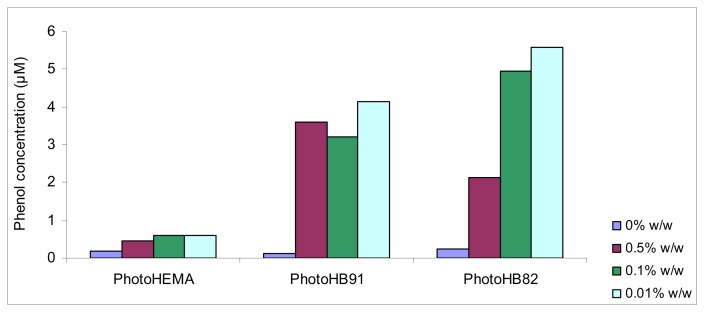
A comparison of the limits of detection of biosensors with various methacrylic-acrylic membranes modified with different amount of gold nanoparticles (Average of n = 5, relative standard deviation RSD = 13-22%).

The effect of gold nanoparticles on the detection appears to follow the trend of the sensitivity slopes of the biosensors (Figure 1). The detection limits became poorer when gold nanoparticles were introduced into the less hydrophilic membranes and the increase in the detection limits of the biosensors with membranes photoHB91 and photoHB82 were statistically significant when compared with no gold nanoparticles was added (significant level α=0.05). But for the phenol biosensor with hydrophilic membrane photoHEMA, there was no significant effect of gold nanoparticles on the detection limits, where little deterioration of the detection limits was observed. The changes in the detection limits were a consequence of the changes in sensitivities. As the sensitivities of the biosensors with photoHB91 and photoHB82 membranes decreased, the detection limits increased. However, the detection limits of these biosensors for phenol are good when compared with other screen printed electrodes based on four electrode system which was reported to be 7.0 μM [14].

### 3.3 Effects of gold nanoparticles on the linear response range for phenol detection

Table 2 shows the linear response range for phenol detection using biosensors based on methacrylic-acrylic membranes without gold nanoparticles or with different amounts of gold nanoparticles added.

In the absence of gold nanoparticle, biosensors with a less hydrophilic membrane such as photoHB82 yielded a narrower linear response range, which was about half of that of the biosensors with more hydrophilic membranes, namely photoHEMA or photoHB91. However, when gold nanoparticles were added to these membranes, the response range to phenol concentrations for all biosensors was extended to higher concentrations, typically more than two times of that without addition of gold nanoparticles. In the case of the more hydrophobic membranes, photoHB91 and photoHB82, the addition of more gold nanoparticles is beneficial as the linear response range of these biosensors increased with the increase in the amount of gold nanoparticles present.

**Figure 2.**
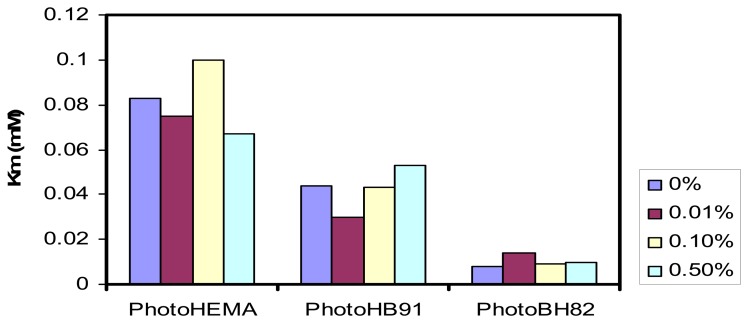
The changes in the Michaelis Menten constants (K_m_) of tyrosinase enzyme immobilized in various methacrylic-acrylic membranes containing different amount of gold nanoparticles. (Number of data used to derive K_m_, n = 17).

The increase in the upper detection range of the phenol biosensors demonstrated that the enzyme affinity for phenol had increased in the present of gold nanoparticles. To examine this further, the Michaelis-Menton constants, K_m_ for enzyme immobilized in various membranes and in the presence of gold nanoparticles was evaluated from the electrochemical Lineweaver-Burk plots using data extracted from the calibration curves of the phenol biosensors. Figure 2 is a comparison of K_m_ values obtained according to different types of methacrylic-acrylic membranes modified with different amounts of gold nanoparticles.

The K_m_ values decrease significantly (significant level α = 0.05) from the more to the less hydrophilic membranes and confirming the increase in affinity of the enzyme to phenol for the less hydrophilic membrane environment. However, the changes in the gold nanoparticle contents of the membranes did not appear to change the k_m_ significantly in each biosensor. A smaller K_m_ value demonstrates that the immobilized tyrosinase possesses higher enzymatic activity. The k_m_ values for all membranes were lower than that found for the free enzyme in solution (0.7 mM) [14]. For tyrosinase immobilized in other reported matrices, the K_m_ values were always lower than that in free solution, e.g. 0.024 mM for tyrosinase immobilized in nano-zeolite/polydialydimethylammonium [2] and 0.14 mM is glassy carbon modified with gold nanoparticles (glutaraldehyde as crosslinker) and 0.0089 mM in graphite-teflon composite modified with gold nanoparticles [4]. For the methacrylic-acrylic matrices investigated here, the values were from 0.008-0.10 mM. Thus, when the tyrosinase is immobilized in a matrix where the diffusion process is restricted, the affinity of the enzyme for the substrate appeared to increase and the K_m_ values decreased. The low K_m_ values may be explained by the fact that *o*-quinone can enter into another enzymatic oxidation, providing a local increase in substrate concentration and an amplification of the electrode response [15].

This shows that the extension in the linear response range of the biosensors is not solely due to a decrease in the K_m_ values or improved affinity of the enzyme to the phenol substrate. It was reported that gold nanoparticles could adsorb redox enzymes (proteins) without loss of their biological activities [9] and the increase in enzyme immobilization via localized adsorption onto the gold nanoparticle may cause a dependent of linear response range of phenol on the amount of gold nanoparticles, especially in the less hydrophilic membranes.

It is likely that both diffusion and the enzyme affinity play an equally important role in controlling the biosensor response in membranes that are more hydrophobic. For example, in the case of phenol biosensor with photoHB82 membrane modified with 0.5% (w/w) and 0.1% (w/w) of gold nanoparticles, the Lineweaver-Burk plot (graph not shown) demonstrated two regimes. The first region was non-linear and observed at low phenol concentrations suggesting diffusion control influence and the second, a linear region occurred at higher substrate concentrations, which suggesting an enzymatic control of the reaction process.

It was observed that the K_m_ values did not demonstrate any good correlation with the sensitivity of the biosensors. Apart from the factor of immobilized enzyme, other factors can affect the biosensor behavior, e.g. inter- and intra-diffusion of substrates and products of reaction, substrate steric and conformational effects, immobilization matrix that may result in enzyme disfiguration, electrode active surface properties that may influence the conductivity, the amount of the enzyme at the surface and the amplification of the biosensor response by recycling process occurs at the electrode surface [16].

### 3.4. Effects of gold nanoparticles on the response time of the biosensors to phenol

**Figure 3.**
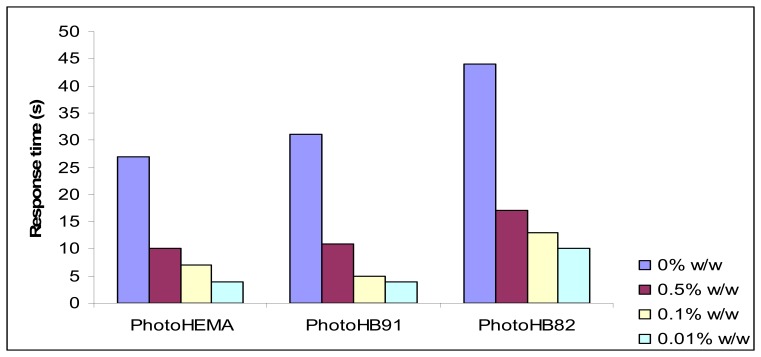
The effect of addition of gold nanoparticles to methacrylic-acrylic membranes on the response time of phenol biosensor. (Average of n = 3 data).

In general biosensor with a more hydrophilic membrane such as photoHEMA demonstrated faster response time compared with biosensor with a less hydrophilic membrane. The difference in response time could be differed by as much as two folds (Figure 3). However, there was a marked (significant level α = 0.05) decrease in response time of all phenol biosensors when gold nanoparticles were added to all types of biosensor membranes. But the changes in the amount of added gold nanoparticles did not change the response time significantly.

A reason why gold nanoparticles may improve the response time of a phenol biosensor is an improvement in the conductivity of the electrode membrane in the presence of such particles. Gold nanoparticles provide a stable surface for enzyme immobilization and can act as electrodes of nano-size that facilitate the electrical communication between enzyme and the bulk electrode materials [4]. It has been widely accepted that gold nanoparticles have some important size-dependent properties due to the quantum size effect. The surface of metallic nanoparticles is always electron deficient and the affinity for electrons will increase with the decrease of dimension [17]. The gold nanoparticles also provide an environment similar to that of the redox protein in a native system and enable the protein molecules more freedom in orientation, thus reducing the insulating property of the protein shells for direct electron transfer and thereby facilitating the electron transfer through some conducting tunnels of gold nanoparticles. In this way, the gold nanoparticles provide the necessary conduction pathways and assist the direct electron transfer between the enzyme and the bulk electrode surface [7]. All of this makes the electron transduction process of the enzymic reaction between phenol and tyrosinase in the membrane to the electrode surface easier, and improves conductivity. Hence a shorter response time was observed for biosensors with all types of membranes.

## 4. Conclusion

The results reported here have shown that the incorporation of gold nanoparticles in the methacrylic-acrylic type of polymer membranes containing tyrosinase can have beneficial effects on the response of biosensors for phenol determination. The most obvious benefits are the improvement in response times and the linear response range of the biosensor. However, incorporation of gold nanoparticles in less hydrophilic membranes has resulted in the reduction in biosensor sensitivity and also yielded poorer detection limits towards phenol. Thus, the hydrophilicity of the membrane matrix where the nanoparticles and enzyme are immobilized plays an important role in influencing the response of the biosensor. For the application of gold nanoparticles in the enhancement of a phenol biosensor response, the nanoparticles should be immobilized in a more hydrophilic matrix rather than a hydrophobic material.

## Figures and Tables

**Table 1.** The linear response slopes of phenol biosensors with photocurable methacrylic-acrylic membranes containing various amounts of gold nanoparticles (50-130 nm) for the determination of phenol.

	PhotoHEMA		PhotoHB91		PhotoHB82	

Gold nano- particles(% w/w)	Slope (μA/μM)	R^2^ (n=17)	Slope (μA/μM)	R^2^ (n=10-14)	Slope (μA/μM)	R^2^ (n=7-12)
0	0.03	0.9769	0.030	0.9946	0.02	0.9789
0.01	0.02	0.9886	0.005	0.9924	0.0007	0.9877
0.1	0.03	0.9912	0.007	0.9847	0.0006	0.9862
0.5	0.03	0.9937	0.008	0.9893	0.0007	0.9900

**Table 2.** Linear response range of phenol that can be detected by methacrylic-acrylic modified gold nanoparticles (50-130 nm).

	Linear concentration range of phenol (μM)		

Gold nanoparticles (% w/w)	PhotoHEMA (n=17)	PhotoHB91 (n=10-14)	PhotoHB82 (n=7-12)
0	6.2 – 42.2	6.2 – 48.2	6.2 – 24.2
0.01	6.2 – 90.2	6.2 – 60.2	6.2 – 42.2
0.1	6.2 – 90.2	6.2 – 66.2	6.2 – 60.2
0.5	6.2 – 90.2	6.2 – 90.2	6.2 – 72.2
